# Extreme Risk of Sudden Cardiac Death within Three Months after Revascularization in Patients with Ischemic Left Ventricular Systolic Dysfunction

**DOI:** 10.31083/j.rcm2410294

**Published:** 2023-10-18

**Authors:** Shaoping Wang, Yi Lyu, Yanci Liu, Shujuan Cheng, Shiying Li, Ze Zheng, Xiaoyan Gu, Ming Gong, Jinghua Liu, Bijan J. Borah

**Affiliations:** ^1^Department of Cardiology, Beijing Anzhen Hospital, Capital Medical University, Beijing Institute of Heart Lung and Blood Vessel Diseases, 100029 Beijing, China; ^2^Department of Health Sciences Research, Mayo Clinic, Rochester, MN 55905, USA; ^3^Department of Anesthesiology, Minhang Hospital, Fudan University, 200240 Shanghai, China; ^4^Department of Echocardiography, Beijing Anzhen Hospital, Capital Medical University, Beijing Institute of Heart Lung and Blood Vessel Diseases, 100029 Beijing, China; ^5^Department of Cardiovascular Surgery, Beijing Anzhen Hospital, Capital Medical University, Beijing Institute of Heart Lung and Blood Vessel Diseases, 100029 Beijing, China; ^6^Robert D. and Patricia E. Kern Center for Science of Health Care Delivery, Mayo Clinic, Rochester, MN 55905, USA

**Keywords:** ejection fraction, heart failure, left ventricular systolic dysfunction, prognosis, revascularization, sudden cardiac death

## Abstract

**Background:**

The risk of sudden cardiac death (SCD) after coronary revascularization in patients with left ventricular (LV) systolic dysfunction has not been characterized completely. This study aims to evaluate the incidence and time course of SCD after revascularization in such patients. The determinants of SCD within 3 months after revascularization were also assessed.

**Methods:**

A cohort study of patients with reduced ejection fraction (EF ≤40%), who underwent revascularization was performed. The incidence of SCD was estimated to account for the competing risk of deaths due to other causes.

**Results:**

2317 patients were enrolled. With a median follow-up of 3.5 years, 162 (32.1%) of the 504 deaths were due to SCD. The risk of SCD was highest in the first 3 months after revascularization, with an incidence rate of 0.37%/month. The event rate decreased to 0.12%/month, 0.08%/month, 0.09%/month, 0.14%/month, and 0.19%/month at 3–6 months, 6–12 months, 1–3 years, 3–5 years, and 5–10 years, respectively. A history of ventricular tachycardia/ventricular fibrillation (hazard ratio [HR], 5.55; 95% confidence interval [CI], 1.33–23.19; *p* = 0.019) and triple vessel disease (HR, 3.90; 95% CI, 1.38–11.05; *p* = 0.010) were associated with the risk of SCD within 3 months. However, preoperative EF (in 5% increments) was not predictive (HR per 5% increase, 0.98; 95% CI, 0.62–1.55; *p *= 0.935).

**Conclusions:**

For patients with LV dysfunction, the risk of SCD was the highest during the first 3 months after revascularization. Further risk classification and treatment strategy are warranted.

**Clinical Trial Registration:**

The name of the registry: Coronary Revascularization in Patients with Ischemic Heart Failure and Prevention of Sudden Cardiac Death. Registration number: ChiCTR2100044378.

## 1. Introduction

Coronary artery disease (CAD) and lower ejection fraction (EF) are two factors associated with sudden cardiac death (SCD) in patients with left ventricular (LV) systolic dysfunction [1]. About 50% of deaths in patients with CAD and LV systolic dysfunction occur suddenly [2, 3]. Attenuating the ischemic state and improving EF with coronary revascularization [4, 5, 6] have been recommended to reduce the risk of SCD [7, 8]. However, the incidence and risk of SCD in patients with CAD and LV dysfunction who underwent coronary revascularization by either percutaneous coronary intervention (PCI) or coronary artery bypass grafting (CABG) have not been well-characterized in the literature.

Current guidelines recommend that patients with LV dysfunction undergo a reassessment of EF 3 months after revascularization to evaluate the neccesity for implantable cardioverter defibrillator (ICD) placement [9]. The rationale for waiting 3 months after revascularization is based on the LV function can improve sufficiently to raise the EF to above 35%, and in clinical trials [10, 11], ICD did not achieve the benefit of SCD prevention early after revascularization (i.e., CABG-patch [12], DINAMIT (Defibrillator in Acute Myocardial Infarction Trial) [13], and IRIS (Immediate Risk stratification Improves Survival trial) [14]). However, how the risk of SCD might develop over time after coronary revascularization is uncertain. Therefore, recognizing the distribution of the incidence of SCD over time, especially within 3 months after revascularization, is clinically significant.

Although EF is currently the most widely used and robust clinical risk factor for SCD after myocardial infarction (MI) and has become the basis for determining a patient’s eligibility for ICD therapy [15, 16], EF might be poor at distinguishing between CAD patients who will die suddenly and those who will die of other cardiovascular causes [2, 17]. Studies in patients with CAD and LV dysfunction who underwent coronary revascularization demonstrated that baseline EF was not associated with the subsequent risk of long-term SCD [18, 19]. This might be due to diverse change of EF after revascularization [7, 20] or requirement of combination with other electrical parameters [2]. Identification of patients at risk remains an issue that is not being adequately addressed.

The present study was designed to determine the incidence of SCD during long-term follow-up in patients with both CAD and LV dysfunction after coronary revascularization by either CABG or PCI, to assess how the event rate changes over time after revascularization, and to analyze clinical predictors of SCD within 3 months after revascularization, especially to understand the predictive value of EF.

## 2. Material and Methods

### 2.1 Study Population

This was a retrospective cohort study with data generated by Beijing Anzhen Hospital. The study protocol was approved by the hospital’s ethics committee. We identified patients’ data with reduced EF (≤40%) who underwent either CABG or PCI from January 2005 to December 2014. Patients were excluded if they had concomitant noncoronary surgery, had no record of coronary angiography, were lost during follow-up. If patients had multiple PCI or CABG during the follow-up, the first qualified procedure was used. The date of the PCI or CABG procedure was considered the index date for analysis.

### 2.2 Data Collection and Definitions

Baseline demographic, clinical, lab test and angiographic parameters were collected from medical records of Beijing Anzhen hospital. The preoperative EF was defined as being measured within 30 days before PCI or CABG. The history of ventricular tachycardia and/or ventricular fibrillation (VT/VF) was determined by prior medical record, electrocardiogram, and 24-hour Holter monitor. Patients with a history of nonsustained VT were also regarded as having a history of VT/VF. Patinets who experienced VT/VF during the acute phase of MI were not regarded as having a history of VT/VF. Electrocardiography at discharge from the index hospitalization for the qualifying procedure was used to diagnose the presence of bundle branch block. Bundle branch block was considered present when the QRS duration was ≥130 ms. Left main disease and triple-vessel disease were defined as severe luminal diamier stenosis in the left main vessel (>50%) or three major epicardial vessels (>70%) by visual assessment. Complete revascularization was defined as successful PCI (residual stenosis of less than 30%) of all angiographically significant lesions (≥70% diameter stenosis) in three coronary arteries and their major branches. For CABG procedures, graft to every primary coronary artery with ≥70% diameter stenosis was accepted as complete revascularization.

### 2.3 Study Outcomes

Outcome data were obtained from medical records at Beijing Anzhen Hospital and through telephone follow up. Death was categorized as cardiac and non-cardiac death. Cardiac death was categorized as SCD and non-SCD [8]. Death due to the procedural and/or acute complication of the revascularization was categorized as non-SCD. Death with insufficient information to make a reasonable decision as to the cause of death was categorized as unknown/unclassified death. According to a modified Hinkle-Thaler system [21], SCD was defined as a sudden, unexpected death that was cardiac in origin, which included those who: (1) died suddenly and unexpectedly within 1 hour of cardiac symptoms in the absence of progressive cardiac deterioration; (2) died unexpectedly in bed during sleep; and (3) died unexpectedly within 24 hours after last being seen alive. For this analysis, the outcome was categorized into 3 groups: patients who died of SCD, patients who died of causes other than SCD, and patients who did not die by the end of the study follow-up period.

### 2.4 Statistical Analysis

Continuous variables were expressed as mean ± SD and categorical variables were expressed as percentages. Baseline characteristics of SCD patients were compared to those of patients who died of other causes, and to patients who were survivors at the end of follow-up. Student’s *t*-test, Rank-sum test, or Chi-square test were used as appropriate for the level of measurement and distribution of the variables.

Given the competing risk of SCD and other modes of deaths, cumulative incidence rates for SCD were estimated with the Fine and Gray method [22]. Cumulative incidence function was fitted using a flexible parametric survival model for competing risks with 3 degrees of freedom for time-dependent effects. SCD, other deaths, and all-cause death rate were summarized with cumulative incidence curves for 10 years of follow-up [23]. Cumulative incidence of SCD in the first year after revascularization was separately displayed along with its 95% confidence interval (CI). Incidence rates per month for SCD were reported at 3 months, 3–6 months, 6–12 months, 1–3 years, 3–5 years and 5–10 years after revascularization.

To identify factors associated with the risk of SCD within 3 months after revascularization, candidate covariates were analyzed in a Cox proportional hazards model by treating death from other causes as a competing risk [22]. The crude associations between the candidate predictors and risk of SCD were first reported by univariate Cox regression. Variables with *p* values ≥ 0.10 were removed from the multivariable model.

All statistical analyses were based on 2-tailed tests. Values of *p* < 0.05 were considered to be statistically significant. Statistical analyses were performed with Stata version 14.0 (StataCorp LP, College Station, TX, USA).

## 3. Results

### 3.1 Baseline Characteristics

Among 2852 initially identified patients, 306 had concomitant noncoronary surgery, 23 had no coronary angiography, and 206 were lost during follow-up. 2317 patients were included in the study, with 1261 (54.4%) undergoing CABG and 1056 (45.6%) undergoinig PCI. There were 1522 (65.7%) patients who were diagnosed with acute coronary syndromes (ACS), which included 535 (23.1%) with ST-segment elevation MI, 179 (7.7%) with non-ST-segment elevation MI, and 808 (34.9%) with unstable angina. There were 795 (34.3%) patients who were diagnosed with stable angina. The mean age was 66.1 years (Table 1). The preoperative EFs were 36.0% (4.5%). There was no patient who had ICD before revascularization. There were 971 patinets who had EF reassessed 3 months after revascularization. The postoperative EFs were 45.3% (11.3%). Among 199 patients whose EFs were ≤35% after revascularization, only 13 (6.5%) patients received ICD (n = 11) or cardiac resynchronization therapy with defibrillation (CRT-D) (n = 2) therapy. Patients with ICD or CRT-D therapy had lower all-cause mortality than did those without ICD or CRT-D therapy (7.7% *vs.* 35.7%, *p* = 0.040) during fowllow-up.

**Table 1.** **Baseline characteristics of the patients**.

Characteristics	SCD (n = 162)	Other deaths (n = 342)	*p* value	No death (n = 1813)	*p* value #
Age (mean ± SD, yr)	70.5 ± 10.4	72.0 ± 9.5	0.108	64.6 ± 10.3	<0.001
Male sex, No. (%)	134 (82.7)	275 (80.4)	0.536	1511 (83.3)	0.838
Hypertension, No. (%)	97 (59.9)	194 (56.7)	0.504	921 (50.8)	0.027
Diabetes, No. (%)	53 (32.7)	133 (38.9)	0.180	614 (33.9)	0.767
eGFR (mean ± SD, mL/min/1.73 $m^{2}$)	74.6 ± 22.3	75.9 ± 23.4	0.571	86.4 (24.6)	<0.001
Cerebral vascular disease, No. (%)	13 (8.0)	50 (14.6)	0.037	166 (9.2)	0.631
History of MI, No. (%)	80 (49.4)	180 (52.6)	0.495	870 (48.0)	0.733
History of VT/VF, No. (%)	6 (3.7)	6 (1.8)	0.180	24 (1.3)	0.018
Atrial fibrillation, No. (%)	13 (8.0)	26 (7.6)	0.868	75 (4.1)	0.022
Bundle branch brock (QRSd ≥130 ms)	8 (4.9)	18 (5.3)	0.878	79 (4.4)	0.730
Preoperative EF (mean ± SD, %)	35.1 ± 5.2	35.2 ± 4.8	0.787	36.3 (4.4)	0.001
ACS, No. (%)	109 (67.3)	242 (70.8)	0.428	1171 (64.6)	0.491
*PCI, No. (%)	75 (46.3)	128 (37.4)	0.058	853 (47.1)	0.854
Triple-vessel disease, No. (%)	93 (59.2)	183 (57.9)	0.783	834 (48.8)	0.012
Left main disease, No. (%)	14 (8.6)	36 (10.8)	0.446	117 (6.5)	0.297
Complete revascularization, No. (%)	89 (54.9)	186 (56.0)	0.820	1028 (57.2)	0.582
Aspirin, No. (%)	141 (92.2)	271 (90.3)	0.522	1703 (94.0)	0.366
Clopidogrel/Ticagrelor, No. (%)	90 (58.8)	173 (57.7)	0.813	1030 (56.8)	0.635
ACEi/ARB/ARNI, No. (%)	74 (48.4)	125 (41.7)	0.174	803 (44.3)	0.333
b-Blocker, No. (%)	113 (73.7)	201 (67.0)	0.135	1444 (79.7)	0.088
MRA, No. (%)	25 (16.3)	44 (14.7)	0.639	292 (16.1)	0.942

*CABG was set as reference to PCI. # *p* values are for the comparison with SCD. Abbreviations: SCD, sudden cardiac death; eGFR, estimated glomerular filtration rate; MI, myocardial infarction; VT/VF, ventricular tachycardia and/or ventricular fibrillation; QRSd, QRS duration; EF, ejection fraction; ACS, acute coronary syndromes; PCI, percutaneous coronary intervention; CABG, coronary artery bypass graft; ACEi, angiotensin-converting enzyme inhibitor; ARB, angiotensin receptor blocker; ARNI, angiotensin receptor-neprilysin inhibitor; MRA, mineralocorticoid receptor antagonist.

After a median follow-up of 3.5 years (interquartile range, 2.0–6.4; maximum = 11.6), 504 (21.8%) patients died during this period. Among those, 162 (32.1%) had SCD, and 342 (67.9%) died from other reasons. The median time to SCD was 3.2 years (interquartile range = 1.0–5.3) after revascularization. Causes of death other than SCD included non-SCD (n = 258), non-cardiac causes of death (n = 79), and unclassified death (n = 5). Non-SCD causes of death included: procedural complications (n = 86); acute MI (n = 59); heart failure (n = 87); other reasons (n = 26). Non-cardiac causes of death included: cancer (n = 30); cerebrovascular accident (n = 24); renal dysfunction (n = 5); pneumonia (n = 2); and other non-cardiac reasons (n = 18). Compared with surviving patients without events, patients who died suddenly were significantly older; were more likely to have a history of hypertension, VT/VF or atrial fibrillation; had a lower estimated glomerular filtration rate (eGFR) and preoperative EF; and were more likely to have triple-vessel diseases. The differences between patients who died of SCD and those who died of other causes were much less significantly different (Table 1).

### 3.2 Incidence of SCD

**Fig. 1.**
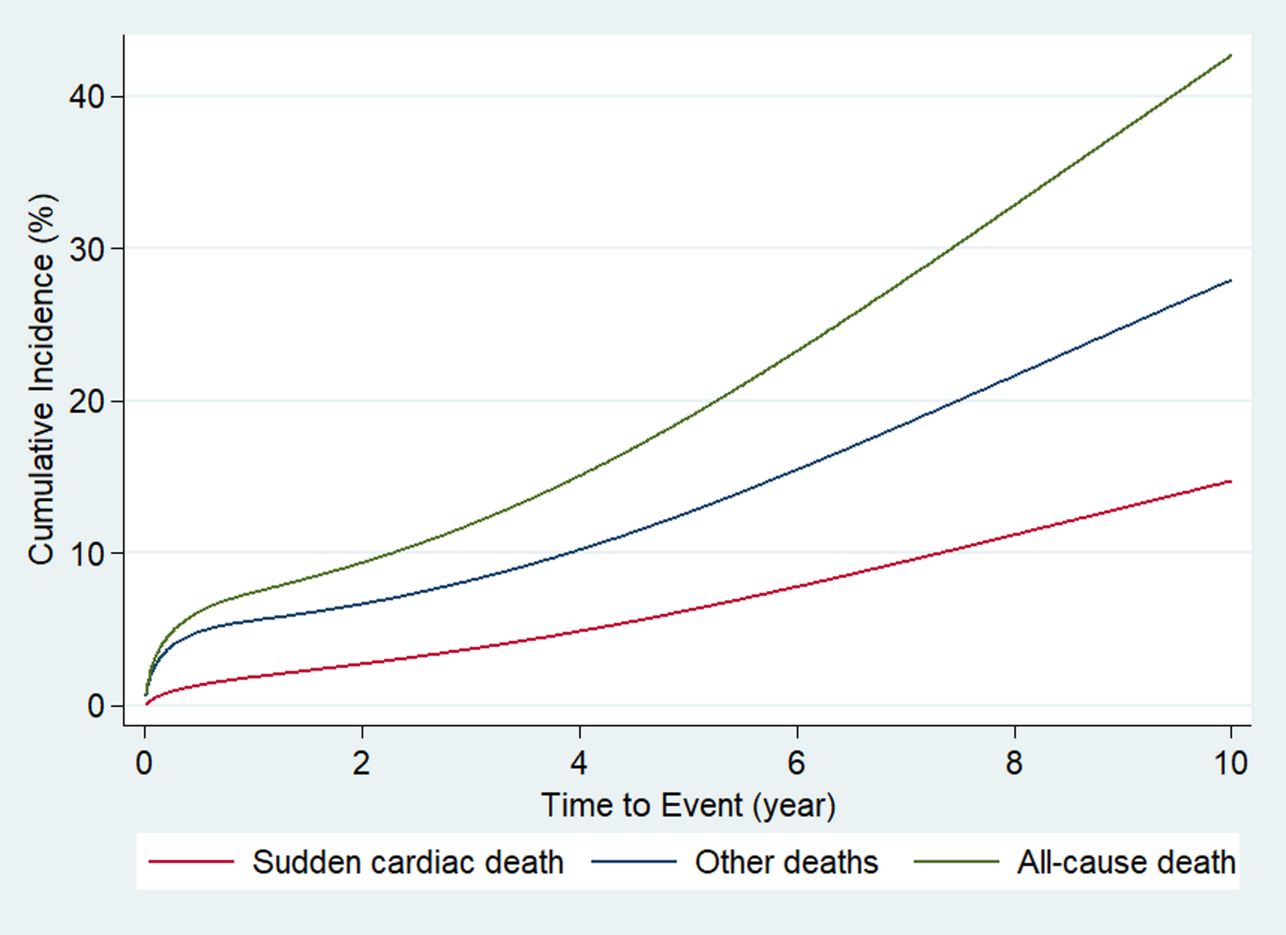
**Cumulative incidence of sudden cardiac death, other deaths and all-cause death after revascularization in patients with coronary artery disease and left ventricular dysfunction**.

**Fig. 2.**
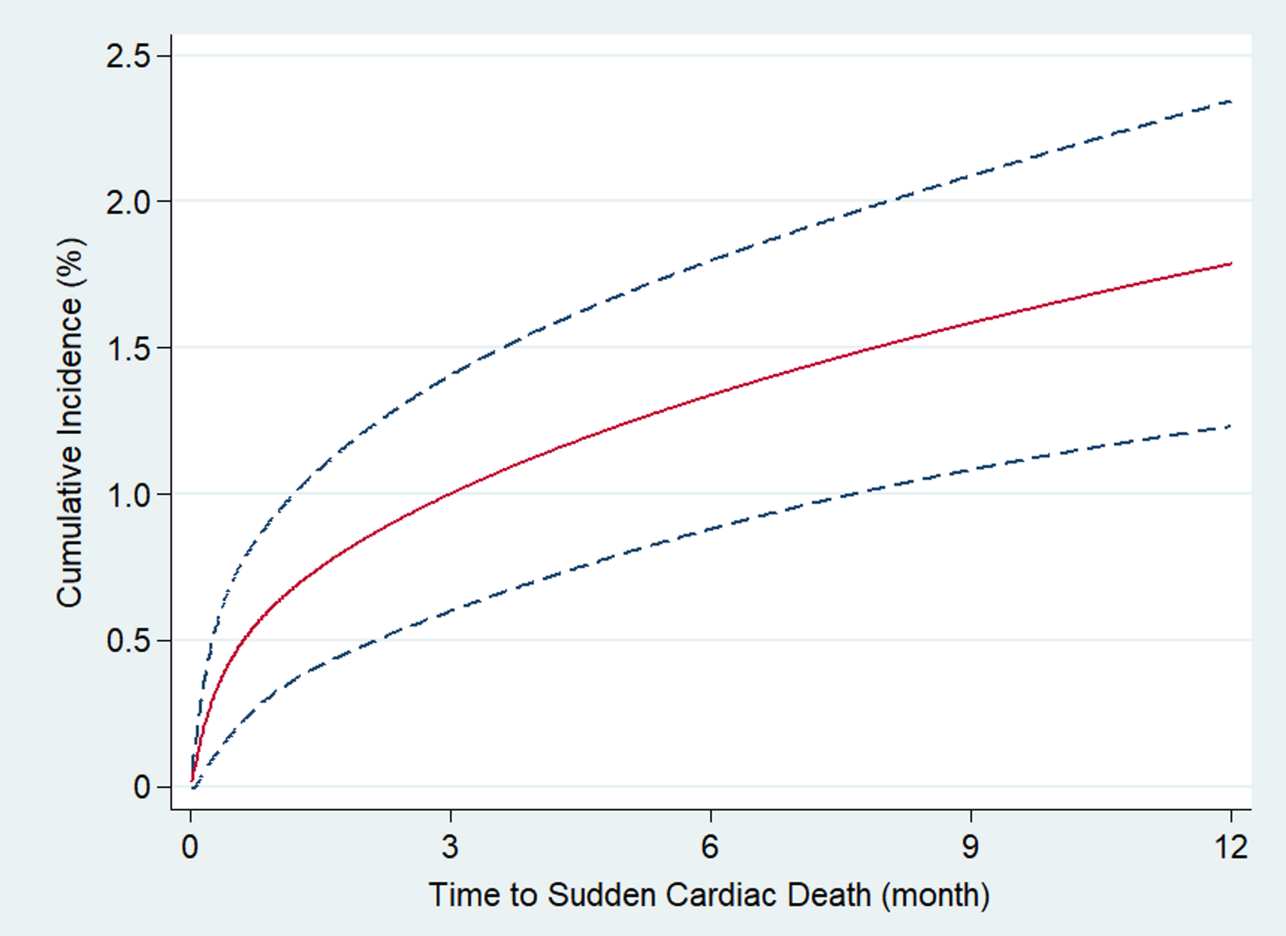
**One-year cumulative incidence of sudden cardiac death after revascularization in patients with coronary artery disease and left ventricular dysfunction.** Dashed lines represented 95% confident intervals.

Fig. 1 shows the cumulative incidence of SCD, other deaths and all-cause death as a function of time after revascularization until 10 years. SCD accounted for one-third of all deaths after revascularization. At 1, 5, and 10 years, the cumulative incidence of SCD was 1.79% (95% CI, 1.23–2.34), 6.40% (95% CI, 5.20–7.59), and 14.67% (95% CI, 12.19–17.15), respectively. Fig. 2 shows the cumulative incidence of SCD within 1 year after revascularization, and demonstrates that the highest event rate was in the first 3 months. There were 23 SCDs in the first 3 months representing 14.2% of all patients with SCD in the study. The incidence rates of SCD/month over time after revascularization are reported in Table 2. The SCD/month in the first 3 months after revascularization was 0.37% (95% CI, 0.25–0.56). Among ACS patients, the SCD/month rate in the first 3 months reached 0.47% (95% CI, 0.30–0.74). After 3 months, the risk/month rate decreased to 0.12% (95% CI, 0.06–0.24) and remained relatively stable thereafter. By the end of the follow-up period, the risk/month rate tended to increase numerically, especially for patients with non-ACS, which had a risk/month rate of 0.25% (95% CI, 0.16–0.39).

**Table 2.** **Incidence rate of sudden cardiac death during follow-up**.

	Time interval after revascularization	Person-month	SCD event, n	Incidence rate per month, % (95% CI)
Total Cohort	3 Months	6221	23	0.37 (0.25–0.56)
	3–6 Months	5998	7	0.12 (0.06–0.24)
	6–12 Months	11,709	9	0.08 (0.04–0.15)
	1–3 Years	41,333	37	0.09 (0.06–0.12)
	3–5 Years	25,095	34	0.14 (0.10–0.19)
	5–10 Years	26,874	50	0.19 (0.14–0.25)
ACS patients	3 Months	4037	19	0.47 (0.30–0.74)
	3–6 Months	3897	3	0.08 (0.02–0.24)
	6–12 Months	7583	8	0.11 (0.05–0.21)
	1–3 Years	26,989	25	0.09 (0.06–0.14)
	3–5 Years	16,181	21	0.13 (0.08–0.20)
	5–10 Years	19,181	31	0.16 (0.11–0.23)
Non-ACS patients	3 Months	2184	4	0.18 (0.07–0.49)
	3–6 Months	2101	4	0.19 (0.07–0.51)
	6–12 Months	4126	1	0.02 (0.00–0.18)
	1–3 Years	14,344	12	0.08 (0.05–0.15)
	3–5 Years	8915	13	0.15 (0.08–0.25)
	5–10 Years	7693	19	0.25 (0.16–0.39)

Abbreviations: SCD, sudden cardiac death; CI, confidence interval; ACS, acute coronary syndrome.

### 3.3 Predictors of SCD within 3 Months

Baseline variables associated with SCD within 3 months are reported in Table 3. In the univariate analysis, history of VT/VF, history of hypertension, and triple-vessel CAD were associated with increased SCD risk. Bundle branch block with QRS duration of ≥130 ms, patients with ACS, and complete revascularization tended to be associated positively with the risk of SCD. In the multivariate model, history of VT/VF (hazard ratio [HR], 5.55; 95% CI, 1.33–23.19; *p *= 0.019), and triple vessel CAD (HR, 3.90; 95% CI, 1.38–11.05; *p *= 0.010) were predictive of SCD. Bundle branch block with a QRS duration of ≥130 ms was marginally significant (HR, 3.39; 95% CI, 1.00–11.50; *p *= 0.050). The cumulative incidence of the first 3 months after revascularization was 5.56% (95% CI, 1.44–21.36) among patients with a history of VT/VF, as compared with 0.97% (95% CI, 0.63–1.48) in patients without a history of VT/VF. Preoperative EF (in 5% increments) was not associated with risk of SCD (HR per 5% increase, 0.98; 95% CI, 0.62–1.55; *p *= 0.935) in the univariate analysis.

**Table 3.** **Baseline Factors Associated with SCD within 3 months in a Multivariate Model**.

Variables	Univariate analysis		Multivariate analysis	
	HR (95% CI)	*p* value	HR (95% CI)	*p* value
Age in 5 years increments	0.99 (0.82–1.19)	0.908		
Male sex	0.48 (0.20–1.16)	0.103		
Hypertension	2.58 (1.02–6.54)	0.046	2.25 (0.88–5.75)	0.091
Diabetes	0.83 (0.34–2.01)	0.673		
eGFR in 5 mL/min increments	0.98 (0.91–1.06)	0.649		
Cerebral vascular disease	0.90 (0.21–3.84)	0.887		
History of MI	1.38 (0.60–3.05)	0.445		
History of anterior MI	1.48 (0.58–3.76)	0.407		
History of VT/VF	6.18 (1.41–27.07)	0.016	5.55 (1.33–23.19)	0.019
Atrial fibrillation	0.86 (0.12–6.33)	0.879		
Bundle branch block (QRSd ≥130 ms)	3.30 (0.98–11.08)	0.054	3.39 (1.00–11.50)	0.050
Preoperative EF in 5% increments	0.98 (0.62–1.55)	0.935		
ACS	2.48 (0.85–7.29)	0.098	2.61 (0.91–7.46)	0.074
Triple-vessel disease	3.56 (1.32–9.57)	0.012	3.90 (1.38–11.05)	0.010
Left main disease	1.99 (0.59–6.71)	0.265		
PCI*	0.60 (0.25–1.41)	0.241		
Complete revascularization	0.49 (0.21–1.13)	0.094	0.80 (0.34–1.90)	0.612
Aspirin	0.73 (0.28–1.87)	0.510		
Clopidogrel/Ticagrelor	0.83 (0.33–2.05)	0.682		
ACEi/ARB/ARNI	1.58 (0.55–4.54)	0.393		
b-Blocker	0.63 (0.20–2.02)	0.441		
MRA	0.39 (0.05–2.95)	0.359		

*CABG was set as reference to PCI. SCD, sudden cardiac death; eGFR, estimated glomerular filtration rate; MI, myocardial infarction; VT/VF, ventricular tachycardia and/or ventricular fibrillation; QRSd, QRS duration; EF, ejection fraction; ACS, acute coronary syndromes; PCI, percutaneous coronary intervention; CABG, coronary artery bypass graft; ACEi, angiotensin-converting enzyme inhibitor; ARB, angiotensin receptor blocker; ARNI, angiotensin receptor-neprilysin inhibitor; MRA, mineralocorticoid receptor antagonist; HR, hazard ratio; CI, confidence interval.

### 3.4 Sensitivity Analysis

The baseline characteristics of patients who underwent CABG and those who underwent PCI are compared in **Supplementary Table 1**. The predictors of SCD for CABG patients and those for PCI patients were explored. Among CABG patients, a history of VT/VF (HR, 9.51; 95% CI, 1.10–82.50; *p *= 0.041) was predictive of SCD in the multivariate analysis (**Supplementary Table 2**). Among PCI patients, a history of VT/VF (HR, 5.05; 95% CI, 1.13–38.62; *p *= 0.038), bundle branch block (HR, 6.24; 95% CI, 1.34–29.04; *p* = 0.020), and triple vessel disease (HR, 4.40; 95% CI, 1.06–18.27; *p *= 0.041) were predictive of SCD (**Supplementary Table 3**).

## 4. Discussion

In the present study, we report three main findings. First, in patients with CAD and EF ≤40% undergoing either PCI or CABG, SCD accounted for one-third of all deaths after revascularization with a 5-year cumulative incidence of 6.40%, and 10-year cumulative incidence of 14.67%. Second, the risk of SCD was highest during the first 3 months after revascularization. Finally, a history of VT/VF or triple vessel CAD was associated with an increased risk of SCD in the 3 months after revascularization, but preoperative EF was not.

In the Multicenter Unsustained Tachycardia Trial (MUSTT) [24], patients with documented CAD, EF ≤40%, and asymptomatic nonsustained VT were included to determine the effect of antiarrhythmic therapy guided by electrophysiological testing. With a follow-up of 5 years, the estimated incidence of SCD among patients with or without antiarrhythmic therapy was 25% and 32%, respectively. Both incidences were much higher than the incidence (6.5% at 5 years) reported in present study, which may indicate the effect of coronary revascularization on reduction of risk of SCD in patients with ischemic LV dysfunction. In addition, in contrast to the MUSTT trial with about 50% arrhythmic deaths, our study had only about one-third SCD. The incidence of SCD reported in the present study was close to the result from the Surgical Treatment for Ischemic Heart Failure (STICH) trial [19], which analyzed a patient sample with preoperative EF ≤35% who underwent CABG and had a 5-year cumulative incidence of 8.5%.

The incident rate per month over different time intervals after revascularization indicated the extreme risk of SCD within 3 months after revascularization, especially for patients with ACS. Similar results had been reported in the STICH tial [19] and VALIANT (Valsartan in Acute Myocardial Infarction Trial) [25]. In the STICH trial, patients enrolled had chronic ischemic heart disease. The SCD/month rate at 1 month, 1–3 months, 3–6 months, 6–12 months, 1–3 years and 3–5years was 0.35%, 0.43%, 0.26%, 0.14%, 0.14%, and 0.11%, respectively. In the VALIANT trial, patients with acute MI complicated by heart failure, LV systolic dysfunction (EF ≤40%), or both, were enrolled. Fewer than 50% of patients had primary PCI or thrombolytic therapy. The incidence of SCD at 1 month, 1–6 months, 6–12 months, 1–2 years and 2–3 years was 1.4%, 0.50%, 0.27%, 0.18% and 0.14%, respectively. Therefore, the period of extreme risk of SCD was early after revascularization.

It is recommended that patients with left ventricular dysfunction undergo a reevaluation of EF 3 months after revascularization for deciding whether to do ICD implantation or not [16]. This interval may allow LV to recover the EF from revascularization. However, the risk of SCD in this period was greatest. Early acute MI also constitutes a period of particularly high risk of death from arrhythmia [25, 26]. The effectiveness of early ICD implantation was explored by the Defibrillator in Acute Myocardial Infarction Trial (DINAMIT) [13] and Immediate Risk-stratification Improves Survival (IRIS) trial [14]. ICD was implanted 6 to 40 days or 5 to 31 days after acute MI, respectively. All the patients had EF ≤35% or EF <40%, assessed within aforementioned intervals, and had been enrolled with additional criteria of impaired cardiac autonomic function or electrical substrate. About 62% to 75% of patients had revascularization by PCI or thrombolysis. ICD failed to reduce overall mortality in both trials, although ICD therapy was associated with a reduction in the rate of SCD. Thus, our data suggests the use of a wearable defibrillator [27] before reassessment of EF 3 months after revascularization, especially for patients with a history of VT/VF or triple vessel disease.

In the present study, a history of VT/VF or bundle branch block was a protential predictor for SCD in the first 3 months after revascularization. Bundle branch block, including both left and right bundle branch blocks, was a powerful and independent predictor of SCD in patients with reduced EF [28] and acute MI [29]. Electrical dispersion of ventricular depolarization and conduction delay, as manifested by QRS prolongation, reflect severity of electrical dysfunction. Bundle branch block with QRS duration ≥130 ms and a history of VT/VF might indicate an arrhythmogenic substrate that is susceptible for arrhythmic death. Electrophysiological abnormality might be more predictable for SCD than is EF [2]. In addition, triple vessel CAD was found to be another factor associated with increased risk of SCD in the 3 months after revascularization, especially for patients who underwent PCI. However, complete revascularization and antiplatelet therapy did not predict the short-term risk of SCD. The potential pathophysiological mechanism needs to be further investigated.

## 5. Limitations

The present study had several limitations. (1) This was an observational study from a single center and thus might have selection bias. (2) An accurate estimate of SCD incidence requires prospective ascertainment of cases. Studies that have used a retrospective death certificate-based method to identify cases of SCD are likely to overestimate [30, 31]. (3) There were 13 patients with ICD implantation during the follow-up, but we did not have data on ICD shocks or aborted sudden cardiac arrest. However, these data were unlikely to have affected the present findings because of their uses were minimal in this cohort. (4) The medical treatments for SCD prevention were underutilized. Both prescripton rate and target-dose achievement did not align with the treatment consensus for SCD prevention, especially for patients who underwent CABG treatment. There were 97.1% who did not achieve the target dose of β-blocker and 97% were below the target dose of angiotensin-converting enzyme inhibitor/angiotensin receptor blocker (ACEI/ARB). One potential reason was that the prescription data were from the time of patient discharge, whereas we analyzed the risk factor of SCD at 3 months after revascularization. The prescription rates of ACEI/ARB/angiotensin receptor-neprilysin inhibitor (ARNI), β-blocker, and mineralocorticoid receptor antagonist (MRA) might have increased during the period of follow-up. (5) Some other factors such as contrast-induced nephropathy [32] were reported as potential risk factors of SCD. However, those data were unavailable in the current study. Multi-center prospective studies are needed to confirm these findings.

## 6. Conclusions 

For patients with CAD and LV systoclic dysfunction who underwent coronary revascularization, SCD accounted for one-third of all deaths. The event rate of SCD was the highest during the first 3 months after revascularization, especially in patients with a history of VT/VF or coronary triple vessel disease. Preoperative EF did not predict the short-term risk of SCD, this underscores the importance of cardiac function surveillance of patients after revascularization.

## Data Availability

The data are available from the corresponding author upon request.

## Supplementary Material

Supplementary material associated with this article can be found, in the online version, at https://doi.org/10.31083/j.rcm2410294.

## References

[1] Shen L, Jhund PS, Petrie MC, Claggett BL, Barlera S, Cleland JGF (2017). Declining Risk of Sudden Death in Heart Failure. *New England Journal of Medicine*.

[2] Buxton AE, Lee KL, Hafley GE, Wyse DG, Fisher JD, Lehmann MH (2002). Relation of ejection fraction and inducible ventricular tachycardia to mode of death in patients with coronary artery disease: an analysis of patients enrolled in the multicenter unsustained tachycardia trial. *Circulation*.

[3] Myerburg RJ, Junttila MJ (2012). Sudden cardiac death caused by coronary heart disease. *Circulation*.

[4] Wang S, Borah BJ, Cheng S, Li S, Zheng Z, Gu X (2021). Diabetes Associated With Greater Ejection Fraction Improvement After Revascularization in Patients With Reduced Ejection Fraction. *Frontiers in Cardiovascular Medicine*.

[5] Wang S, Lyu Y, Cheng S, Zhang Y, Gu X, Gong M (2022). Smaller left ventricular end-systolic diameter and lower ejection fraction at baseline associated with greater ejection fraction improvement after revascularization among patients with left ventricular dysfunction. *Frontiers in Cardiovascular Medicine*.

[6] Wang S, Cheng S, Zhang Y, Lyu Y, Liu J (2022). Extent of Ejection Fraction Improvement After Revascularization Associated with Outcomes Among Patients with Ischemic Left Ventricular Dysfunction. *International Journal of General Medicine*.

[7] Vakil K, Florea V, Koene R, Kealhofer JV, Anand I, Adabag S (2016). Effect of Coronary Artery Bypass Grafting on Left Ventricular Ejection Fraction in Men Eligible for Implantable Cardioverter-Defibrillator. *American Journal of Cardiology*.

[8] Carson P, Wertheimer J, Miller A, O’Connor CM, Pina IL, Selzman C (2013). The STICH trial (Surgical Treatment for Ischemic Heart Failure): mode-of-death results. *JACC: Heart Failure*.

[9] Kusumoto FM, Calkins H, Boehmer J, Buxton AE, Chung MK, Gold MR (2014). HRS/ACC/AHA expert consensus statement on the use of implantable cardioverter-defibrillator therapy in patients who are not included or not well represented in clinical trials. *Circulation*.

[10] Moss AJ, Zareba W, Hall WJ, Klein H, Wilber DJ, Cannom DS (2002). Prophylactic implantation of a defibrillator in patients with myocardial infarction and reduced ejection fraction. *New England Journal of Medicine*.

[11] Moss AJ, Hall WJ, Cannom DS, Daubert JP, Higgins SL, Klein H (1996). Improved survival with an implanted defibrillator in patients with coronary disease at high risk for ventricular arrhythmia. Multicenter Automatic Defibrillator Implantation Trial Investigators. *New England Journal of Medicine*.

[12] Bigger JT (1997). Prophylactic use of implanted cardiac defibrillators in patients at high risk for ventricular arrhythmias after coronary-artery bypass graft surgery. Coronary Artery Bypass Graft (CABG) Patch Trial Investigators. *New England Journal of Medicine*.

[13] Hohnloser SH, Kuck KH, Dorian P, Roberts RS, Hampton JR, Hatala R (2004). Prophylactic use of an implantable cardioverter-defibrillator after acute myocardial infarction. *New England Journal of Medicine*.

[14] Steinbeck G, Andresen D, Seidl K, Brachmann J, Hoffmann E, Wojciechowski D (2009). Defibrillator implantation early after myocardial infarction. *New England Journal of Medicine*.

[15] Ponikowski P, Voors AA, Anker SD, Bueno H, Cleland JGF, Coats AJS (2016). 2016 ESC Guidelines for the diagnosis and treatment of acute and chronic heart failure: The Task Force for the diagnosis and treatment of acute and chronic heart failure of the European Society of Cardiology (ESC)Developed with the special contribution of the Heart Failure Association (HFA) of the ESC. *European Heart Journal*.

[16] Yancy CW, Jessup M, Bozkurt B, Butler J, Casey DE, Drazner MH (2013). 2013 ACCF/AHA guideline for the management of heart failure: executive summary: a report of the American College of Cardiology Foundation/American Heart Association Task Force on practice guidelines. *Circulation*.

[17] Every N, Hallstrom A, McDonald KM, Parsons L, Thom D, Weaver D (2002). Risk of sudden versus nonsudden cardiac death in patients with coronary artery disease. *American Heart Journal*.

[18] Daoud EG, Niebauer M, Kou WH, Man KC, Horwood L, Morady F (1995). Incidence of implantable defibrillator discharges after coronary revascularization in survivors of ischemic sudden cardiac death. *American Heart Journal*.

[19] Rao MP, Al-Khatib SM, Pokorney SD, She L, Romanov A, Nicolau JC (2017). Sudden Cardiac Death in Patients With Ischemic Heart Failure Undergoing Coronary Artery Bypass Grafting: Results From the STICH Randomized Clinical Trial (Surgical Treatment for Ischemic Heart Failure). *Circulation*.

[20] Adachi Y, Sakakura K, Wada H, Funayama H, Umemoto T, Fujita H (2016). Determinants of Left Ventricular Systolic Function Improvement Following Coronary Artery Revascularization in Heart Failure Patients With Reduced Ejection Fraction (HFrEF). *International Heart Journal*.

[21] Greenberg H, Case RB, Moss AJ, Brown MW, Carroll ER, Andrews ML (2004). Analysis of mortality events in the Multicenter Automatic Defibrillator Implantation Trial (MADIT-II). *Journal of the American College of Cardiology*.

[22] Fine JP, Gray RJ (1999). A Proportional hazards model for the subdistribution of a competing risk. *Journal of the American Statistical Association*.

[23] Hinchliffe SR, Lambert PC (2013). Extending the flexible parametric survival model for competing risks. *Stata Journal*.

[24] Buxton AE, Lee KL, Fisher JD, Josephson ME, Prystowsky EN, Hafley G (1999). A randomized study of the prevention of sudden death in patients with coronary artery disease. Multicenter Unsustained Tachycardia Trial Investigators. *New England Journal of Medicine*.

[25] Solomon SD, Zelenkofske S, McMurray JJV, Finn PV, Velazquez E, Ertl G (2005). Sudden death in patients with myocardial infarction and left ventricular dysfunction, heart failure, or both. *New England Journal of Medicine*.

[26] Adabag AS, Therneau TM, Gersh BJ, Weston SA, Roger VL (2008). Sudden death after myocardial infarction. *Journal of the American Medical Association*.

[27] Piccini JP, Allen LA, Kudenchuk PJ, Page RL, Patel MR, Turakhia MP (2016). Wearable Cardioverter-Defibrillator Therapy for the Prevention of Sudden Cardiac Death: A Science Advisory From the American Heart Association. *Circulation*.

[28] Hawkins NM, Wang D, McMurray JJV, Pfeffer MA, Swedberg K, Granger CB (2007). Prevalence and prognostic impact of bundle branch block in patients with heart failure: evidence from the CHARM programme. *European Journal of Heart Failure*.

[29] Vivas D, Pérez-Vizcayno MJ, Hernández-Antolín R, Fernández-Ortiz A, Bañuelos C, Escaned J (2010). Prognostic implications of bundle branch block in patients undergoing primary coronary angioplasty in the stent era. *American Journal of Cardiology*.

[30] Every NR, Parsons L, Hlatky MA, McDonald KM, Thom D, Hallstrom AP (1997). Use and accuracy of state death certificates for classification of sudden cardiac deaths in high-risk populations. *American Heart Journal*.

[31] Chugh SS, Jui J, Gunson K, Stecker EC, John BT, Thompson B (2004). Current burden of sudden cardiac death: multiple source surveillance versus retrospective death certificate-based review in a large U.S. community. *Journal of the American College of Cardiology*.

[32] Garcia S, Ko B, Adabag S (2012). Contrast-induced nephropathy and risk of acute kidney injury and mortality after cardiac operations. *Annals of Thoracic Surgery*.

