# Supplementary material for: Extreme Risk of Sudden Cardiac Death within Three Months after Revascularization in Patients with Ischemic Left Ventricular Systolic Dysfunction
Source: Rev Cardiovasc Med. 2023 Oct 18;24(10):294. doi: 10.31083/j.rcm2410294 (PMC11273152; doi:10.31083/j.rcm2410294)
Supplement: Supplementary file 1 [file 2153-8174-24-10-294-s1.docx]

**Supplemental Table 1. Comparison of baseline characteristics between patients who underwent PCI versus CABG ^a^**

| Characteristic | All Patients  (N=2,317) | PCI  (N=1,056) | CABG  (N=1,261) | P Value |
| --- | --- | --- | --- | --- |
| Age, y | 66.1 (10.6) | 65.7 (11.6) | 66.4 (9.8) | 0.128 |
| Male sex | 1,902 (82.9) | 852 (80.7) | 1,068 (84.7) | 0.011 |
| Hypertension | 1,212 (52.3) | 587 (55.6) | 625 (49.6) | 0.004 |
| Diabetes | 800 (34.5) | 336 (31.8) | 464 (36.8) | 0.012 |
| eGFR, mL/min/1.73m^2^ | 84.1 (24.7) | 84.8 (23.6) | 83.5 (25.6) | 0.221 |
| Cerebral vascular disease | 229 (9.9) | 94 (8.9) | 135 (10.7) | 0.147 |
| History of MI | 1,130 (48.8) | 466 (44.1) | 664 (52.7) | <0.001 |
| History of anterior MI | 441 (19.0) | 255 (24.2) | 186 (14.8) | <0.001 |
| History of VT/VF | 36 (1.6) | 31 (2.9) | 5 (0.4) | <0.001 |
| Atrial fibrillation | 114 (4.9) | 59 (5.6) | 55 (4.4) | 0.174 |
| Bundle branch block (QRSd≥130ms) | 105 (4.5) | 56 (5.3) | 49 (3.9) | 0.102 |
| Preoperative EF | 36.0 (4.5) | 35.9 (4.7) | 36.1 (4.3) | 0.227 |
| ACS | 1,522 (65.7) | 852 (80.7) | 670 (53.1) | <0.001 |
| Triple-vessel disease | 1,110 (50.9) | 278 (29.2) | 832 (67.6) | <0.001 |
| Left main disease | 167 (7.3) | 52 (5.0) | 115 (9.2) | <0.001 |
| Complete revascularization | 1,303 (56.9) | 412 (39.4) | 891 (71.5) | <0.001 |
| Aspirin | 2,099 (92.7)) | 1,024 (98.0) | 1,075 (88.1) | <0.001 |
| Clopidogrel/Ticagrelor | 1,493 (65.9) | 1,026 (98.2) | 467 (38.3) | <0.001 |
| ACEi/ARB/ARNI | 1,002 (44.2) | 690 (66.00 | 312 (25.6) | <0.001 |
| β-Blocker | 1,758 (77.6) | 834 (79.8) | 924 (75.7) | 0.020 |
| MRA | 361 (15.9) | 238 (22.8) | 123 (10.1) | <0.001 |

^a^ Values are mean (SD) or No. of patients (%).

Supplemental Table 2. Baseline Factors Associated with SCD within 3 months after CABG

| Variables | Univariate analysis | | Multivariate analysis | |
| --- | --- | --- | --- | --- |
|  | HR(95%CI) | P value | HR(95%CI) | P value |
| Age in 5 years increments | 0.99(0.94-1.05) | 0.811 |  |  |
| Male sex | 0.51(0.16-1.58) | 0.242 |  |  |
| Hypertension | 2.04(0.70-5.98) | 0.192 |  |  |
| Diabetes | 0.62(0.20-1.93) | 0.404 |  |  |
| eGFR in 5 mL/min increments | 1.00(0.98-1.01) | 0.610 |  |  |
| Cerebral vascular disease | 0.63(0.08-4.80) | 0.656 |  |  |
| History of MI | 1.83(0.62-5.35) | 0.271 |  |  |
| History of anterior MI | 2.89(0.99-8.44) | 0.053 | 2.47(0.85-7.18) | 0.097 |
| History of VT/VF | 20.38(2.32-179.31) | 0.007 | 9.51(1.10-82.50) | 0.041 |
| Atrial fibrillation | Not applicable | |  |  |
| Bundle branch block (QRSd≥130ms) | 1.89(0.25-14.37) | 0.541 |  |  |
| Preoperative EF in 5% increments | 0.97(0.87-1.07) | 0.549 |  |  |
| ACS | 3.66(1.04-12.89) | 0.043 | 3.30(0.92-11.80) | 0.066 |
| Triple-vessel disease | 3.09(0.70-13.70) | 0.137 |  |  |
| Left main disease | 1.59(0.36-7.03) | 0.539 |  |  |
| Complete revascularization | 0.44(0.16-1.21) | 0.111 |  |  |
| Aspirin | 0.49(0.14-1.75) | 0.271 |  |  |
| Clopidogrel/Ticagrelor | 1.26(0.44-3.60) | 0.672 |  |  |
| ACEi/ARB/ARNI | 1.94(0.55-6.85) | 0.306 |  |  |
| β-Blocker | 0.47(0.13-1.65) | 0.238 |  |  |
| MRA | Not applicable | |  |  |

Supplemental Table 3. Baseline Factors Associated with SCD within 3 months after PCI

| Variables | Univariate analysis | | Multivariate analysis | |
| --- | --- | --- | --- | --- |
|  | HR(95%CI) | P value | HR(95%CI) | P value |
| Age in 5 years increments | 1.00(0.95-1.06) | 0.951 |  |  |
| Male sex | 0.40(0.10-1.66) | 0.207 |  |  |
| Hypertension | 5.60(0.69-45.46) | 0.107 |  |  |
| Diabetes | 1.29(0.31-5.38) | 0.730 |  |  |
| eGFR in 5 mL/min increments | 1.00(0.97-1.03) | 0.969 |  |  |
| Cerebral vascular disease | 1.46(0.18-11.99) | 0.723 |  |  |
| History of MI | 0.75(0.18-3.15) | 0.700 |  |  |
| History of anterior MI | 0.45(0.06-3.64) | 0.452 |  |  |
| History of VT/VF | 4.91(1.05-46.47) | 0.042 | 5.05(1.13-38.62) | 0.038 |
| Atrial fibrillation | 2.38(0.30-19.17) | 0.414 |  |  |
| Bundle branch block (QRSd≥130ms) | 6.13(1.25-30.11) | 0.026 | 6.24(1.34-29.04) | 0.020 |
| Preoperative EF in 5% increments | 1.04(0.86-1.27) | 0.657 |  |  |
| ACS | 1.68(0.21-13.60) | 0.629 |  |  |
| Triple-vessel disease | 4.07(0.98-16.99) | 0.054 | 4.40(1.06-18.27) | 0.041 |
| Left main disease | 2.74(0.34-22.16) | 0.345 |  |  |
| Complete revascularization | 0.22(0.03-1.78) | 0.155 |  |  |
| Aspirin | 1.39(0.31-6.21) | 0.664 |  |  |
| Clopidogrel/Ticagrelor | 0.27(0.03-2.25) | 0.227 |  |  |
| ACEi/ARB/ARNI | Not applicable | |  |  |
| β-Blocker | Not applicable | |  |  |
| MRA | 1.13(0.12-10.87) | 0.916 |  |  |
